# The effectiviness of an innovative web-based application to monitor HRQOL problems in paediatric rheumatology clinical practice

**DOI:** 10.1186/1546-0096-9-S1-P128

**Published:** 2011-09-14

**Authors:** L Haverman, MAJ van Rossum, HSA Heymans, TW Kuijpers, MA Grootenhuis

**Affiliations:** 1 Academic Medical Center / Emma Children’s Hospital, Pediatric Psychosocial Department, Amsterdam, Netherlands; 2 Academic Medical Center / Emma Children’s Hospital, Department of Pediatric Rheumatology and Immunology, Amsterdam, Netherlands; 3 Reade (location Jan van Breemen), Department of Pediatric Rheumatology, Amsterdam, Netherlands

## Introduction

The use of Patient Reported Outcomes (PROs) in daily clinical practice receives increasing attention. Former studies suggest that discussing PROs improves communication between physicians and patients and facilitates early recognition of HRQOL problems. Especially in paediatrics, there is a need to address HRQOL in daily clinical practice in the context of a child’s development; repeated measurement of HRQOL in different developmental stages can be valuable. With the use of PROs, HRQOL problems can be detected early and tailored intervention can be provided to the child, before the HRQOL problems increase. However research of PROs in paediatrics is still scarce. Children with Juvenile Idiopathic Arthritis (JIA) are at risk for HRQOL problems. Therefore, it is important to monitor their HRQOL during doctor’s visits. The aim of this study is to investigate the effectiveness of a web-based PRO about HRQOL (KLIK PROfile) in paediatric rheumatology practice.

## Material and methods

Children with JIA participated in a sequential cohort intervention study. A control group without the use of the KLIK PROfile was compared to an intervention group with the use of the KLIK PROfile. Outcomes of effectiveness were HRQOL topics discussed, satisfaction and opinion about the KLIK PROfile.

## Results

176 (64.5%) children participated. The KLIK PROfile increased discussion about psychosocial topics (p<.05). Additionally, the paediatrician’s satisfaction about the consultation increased (p<.01). The KLIK PROfile did not influence the parents’ satisfaction. The parents rated the use of the KLIK PROfile with an 8 (median). The paediatricians regarded the PROfile meaningful in 90.2 % of the consultations.

## Conclusion

We conclude that a web-based application to systemically monitor HRQOL problems in paediatric clinical practice is effective and should be implemented.

